# Effect of lipid-based nutrient supplement—Medium quantity on reduction of stunting in children 6-23 months of age in Sindh, Pakistan: A cluster randomized controlled trial

**DOI:** 10.1371/journal.pone.0237210

**Published:** 2020-08-13

**Authors:** Gul Nawaz Khan, Sumra Kureishy, Shabina Ariff, Arjumand Rizvi, Muhammad Sajid, Cecilia Garzon, Ali Ahmad Khan, Saskia de Pee, Sajid Bashir Soofi, Zulfiqar A. Bhutta

**Affiliations:** 1 Department of Paediatrics and Child Health, Aga Khan University, Karachi, Pakistan; 2 World Food Programme, Islamabad, Pakistan; 3 World Food Programme, Rome, Italy; 4 Friedman School of Nutrition Science and Policy, Tufts University, Boston, MA, United States of America; 5 Division of Human Nutrition, Wageningen University, Wageningen, Netherlands; 6 Centre of Excellence in Women and Child Health, Aga Khan University, Karachi, Pakistan; Institut de recherche pour le developpement, FRANCE

## Abstract

**Background:**

Chronic childhood malnutrition, or stunting, remains a persistent barrier to achieve optimal cognitive development, child growth and ability to reach full potential. Almost half of children under-five years of age are stunted in the province of Sindh, Pakistan.

**Objective:**

The primary objective of this study was to test the hypothesis that the provision of lipid-based nutrient supplement—medium-quantity (LNS-MQ) known as Wawamum will result in a 10% reduction in risk of being stunted at the age of 24 months in the intervention group compared with the control group.

**Design:**

A cluster randomized controlled trial was conducted in Thatta and Sujawal districts of Sindh province, Pakistan. A total of 870 (419 in intervention; 451 in control) children between 6–18 months old were enrolled in the study. The unit of randomization was union council and considered as a cluster. A total of 12 clusters, 6 in each study group were randomly assigned to intervention and control group. All children received standard government health services, while children in the intervention group also received 50 grams/day of Wawamum.

**Results:**

Children who received Wawamum were found to have a significantly reduced risk of stunting (RR = 0.91, 95% CI; 0.88–0.94, p<0.001) and wasting (RR = 0.78, 95% CI; 0.67–0.92, p = 0.004) as compared to children who received the standard government health services. There was no evidence of a reduction in the risk of underweight (RR = 0.94, 95% CI; 0.85–1.04, p = 0.235) in the intervention group compared to the control group. Statistically significant reduction in anaemia in the intervention group was also found as compared to the control group (RR = 0.97, 95% CI; 0.94–0.99, p = 0.042). The subgroup analysis by age, showed intervention effect is significant in reduction of risk of stunting in younger children of aged 6–12 month (RR = 0.83, 95% CI; 0.81–0.86, p = <0.001) and their older peers aged 13–18 month- (RR = 0.90, 95% CI; 0.83–0.97, p = 0.008). The mean compliance of Wawamum was 60% among children.

**Conclusions:**

The study confirmed that the provision of Wawamum to children 6–23 months of age is effective in reducing the risk of stunting, wasting and anaemia. This approach should be scaled up among the most food insecure areas/households with a high prevalence of stunting to achieve positive outcomes for nutrition and health. This study was registered at clinicaltrials.gov as NCT02422953.

Clinical Trial Registration Number: NCT02422953

## Introduction

Global progress to reduce stunting among children under-five is not rapid enough to meet the World Health Assembly target to reduce by 40% the number of children who are stunted by 2025 [1]. Nearly 155 million or 23% of children in the world are affected by stunting. It is well known that stunted children are a high risk of mortality, lower academic achievements, low economic productivity, reduced cognition, higher risk of morbidities also in adulthood, as well as a major contribution to the intergenerational cycle of undernutrition and poverty [2–8].

In 2016, two of every five of the world’s stunted children and more than half of all wasted children lived in South Asia [9]. In Pakistan, stunting is a major public health problem. The 2017–18 Pakistan Demographic and Health Survey (PDHS) reported that 38% of children were stunted and 17% were severely stunted [10]. The survey also found that stunting was inversely related to wealth; 57% of children in the lowest wealth quintile were stunted as compared to only 22% of children in the highest wealth quintile. The causes of sub-optimum child growth and development in low-income countries are inadequate dietary intake, poor maternal and child health, household food insecurity, inadequate care practices, unhealthy household environment and a diet of mainly plant-based foods, with limited intake of animal products, diary and legumes [11, 12]. Community-based interventions targeting complementary feeding are usually focused on children 6–23 months of age as they are more susceptible to a high incidence of growth faltering, micronutrient deficiencies and infectious illnesses in developing countries [11, 13]. Therefore, interventions that provide fortified complementary food supplements have the potential to improve both macronutrient and micronutrient intake in children [14–19]. A recent review shows weight (mean difference 0.12 kg; 95% CI 0.05–0.18) and height gains (mean difference 0.27 cm; 95% CI 0.07–0.48) through supplementation in children under five living in food insecure and vulnerable settings [20].

Medium-quantity lipid-based nutrient supplements (LNS-MQ) were developed for prevention of undernutrition. The guidelines on the use of LNS for prevention of undernutrition is mostly based on programme circumstances and availability of commodities and highlights the needs for more evidence about impact of the different supplements on nutritional status in specific circumstances [21, 22]. Studies conducted in Ghana and Malawi suggested that consumption of LNS might boost length gain and reduce growth failure and the incidence of severe stunting among 6-18-month-old infants [23–25].

Evidence suggests that behavioral change interventions are also effective in improving infant and young child feeding (IYCF) practices. However, evidence on the impact of behavioral change interventions on anthropometric measurements is mixed, from reports of no change to significant changes in child growth indicators, depending on the intervention design, length of intervention and study setting [26–28]. Evidence on LNS-MQ impacting child stunting is lacking in community-based studies. The primary objective of this study was to test the hypothesis that a locally produced LNS-MQ, known as Wawamum [29], delivered through the lady health workers (LHWs) programme, is successful in eliciting a 10% reduction in risk of stunting in children 6–23 months of age.

## Methods

### Study design

This was a cluster randomized controlled trial (RCT) conducted from August 2014 to December 2016 in Thatta and Sujawal districts of Sindh, Pakistan. Three cohorts were enrolled in the study to assess the impact of the nutrition-based interventions on stunting. The first cohort consisted of pregnant and lactating women (PLW), who received wheat soya blend (WSB) during pregnancy and the first six months of lactation. As part of the first cohort, children born to the PLW were also followed till the child reached 24 months. The second cohort consisted of children aged 6–23 months who received Wawamum, while the third cohort of children 24–59 months received micro-nutrient powder (MNP). This article only presents the results of second cohort of the study, children 6–23 months of age.

### Study participants and inclusion criteria

For second cohort, households were considered eligible if they had a child between 6–18 months old at the time of enrolment, so that a minimum of six months of intervention exposure for each child. Study enrolment occurred on a rolling basis from August 2014 to June 2016. The LHW family register was used to identify households with children 6–18 months.

### Interventions

A locally produced lipid-based nutrient supplement—medium quantity (LNS-MQ), known as Wawamum (consists of roasted chickpeas, vegetable oil, dry skimmed milk powder, sugar, micronutrients, emulsifier and antioxidant) was distributed amongst children aged 6–23 months. A daily ration of 50 grams of Wawamum was provided during 6–23 months of age to cover the RDA of most micronutrients and a minimum of 255 kcal of energy (about 1/4 of daily energy requirements for children in this age range), Table 1 [29]. Government Lady Health Workers (LHWs), a vital part of the government’s primary healthcare system, delivered the interventions and also provided health and hygiene messages to the intervention group. The control group received routine public and private health services available within the area. These public health services were provided by Basic Health Unit (BHUs), Rural Health Centers (RHCs), Taluka Hospitals, District Headquarter Hospital (DHQ) and local healers available in the community.

**Table 1:** Composition of nutrient values in LNS-MQ supplement.

Nutrients values per 50 g product (one serving)	Unit	Minimum	Maximum
Energy	Kcal	255	280
Protein	g	5.5	8
Fat	g	13	18
ω-3 fatty acids	g	0.15	0.9
ω-6 fatty acid	g	1.3	3.1
Retinol (Vitamin A)	mcg	275	575
Thiamin (Vitamin B1)	mg	0.5	-
Riboflavin (Vitamin B2)	mg	1.05	-
Niacin (Vitamin B3)	mg	6.5	-
Pantothenic Acid (Vitamin B5)	mg	2	-
Pyridoxine (Vitamin B6)	mg	0.9	-
Biotin (Vitamin B7)	mcg	30	-
Folate (Vitamin B9) DFE	mcg	165	-
Cobalamin (Vitamin B12)	mcg	1	-
Ascorbate (Vitamin C)	mg	30	-
Cholecalciferol (Vitamin D)	mcg	7.5	10
Tocopherol Acetate (Vitamin E)	mg aTE	8	-
Phytomenadione (Vitamin K)	mcg	13.5	-
Calcium (Ca)	mg	268	375
Copper (Cu)	mg	0.7	1.0
Iodine (I)	mcg	50	70
Iron (Fe)	mg	5	7
Magnesium (Mg)	mg	75	113
Manganese (Mn)	mg	0.6	1.2
Phosphorus (P)	mg	225	375
Potassium (K)	mg	450	700
Selenium (Se)	mcg	10	20
Sodium (Na)	mg	-	135
Zinc (Zn)	mg	5.5	7

### Outcomes

#### Primary outcome

The primary outcome was stunting, defined as length-for-age <-2 standard deviation (SD) from the median of the World Health Organization (WHO) child growth standards in intervention group compared with control group.

#### Secondary outcome

The secondary outcomes were wasting and underweight, defined as weight-for-length <-2 SD and weight-for-age <-2 SD from the median of the WHO child growth standards and anemia (<11gm/dl) in intervention group compared with control group.

### Sample size

The sample size was calculated to detect a 10% difference in stunting prevalence in the intervention group compared to the control group. We used the 50% stunting prevalence in Sindh from the National Nutrition Survey (NNS 2011) as a baseline to estimate the sample size. We calculated a sample size of 70–75 children per cluster with a statistical significance of 0.05, power of 0.80, and an intra-cluster correlation of 0.0025 [30]. This led to a final sample size of 12 clusters with 870 children 6–18 months in the intervention and control groups to be enrolled into the trial.

### Randomization

The unit of randomization was a union council (UC) with a population of 25,000–30,000. UC is smallest administrative unit in Pakistan. Each UC has at least one public-sector healthcare facility and 15–20 LHWs affiliated with the facility. Of the 29 UCs, where the stunting prevention programme was implemented, 12 UCs were randomly allocated to intervention and control groups with a computer generated randomization sequence that was generated by an independent expert at the Data Management Unit in Aga Khan University, Pakistan. Clusters were matched based on the stunting rate in children under five years and population size.

### Data collection

Six data collection teams received training for six days on data collection techniques, anthropometric measurements, haemoglobin testing, ethical issues and data collection tools. Each team consisted of 4 female data collectors and 1 male team leader. After enrolment of study participants, the teams conducted monthly follow-up to assess compliance to the intervention and child morbidity and mortality from September 2014 to December 2016. Data on compliance was collected using parental recall and observation of used and unused sachets at household level. Anthropometric data was collected on a quarterly basis to monitor the linear growth and weight gain of children and its association with the intervention. The length and weight of children were measured through the Seca anthropometry kits.

A separate verification team randomly visited 5% of the households assessed by the data collection team to validate the data quality. The data collection process was also supervised and monitored by team leaders, a field supervisor and a study manager. ENA-SMART software was used to track and conduct plausibility checks for anthropometric measurements taken by the field supervisor on a weekly and by the study manager on a monthly basis.

### Data analysis

Analysis was conducted using intention-to-treat approach. Proportions, means (with standard deviations), and medians (with interquartile ranges) are presented for baseline variables. Pearson Chi-square test was used to establish an association between categorical variables and Student’s t-tests were used for continuous data. The anthropometric measures together with the age and sex of the children were used to calculate the weight-for-age (underweight), height-for-age (stunting) and weight-for-height (wasting) z-scores according to the WHO growth standards [31]. Relative risk of stunting, wasting, and underweight in children 6–23 months were compared between intervention and control groups using generalized linear model model (GLM) to adjust for clustering effect. The log (link)function is used for binary outcomes. The analysis further adjusted for baseline nutritional status and duration of exposure to the intervention Anaemia was defined as haemoglobin levels <11gm/dl. P-values below 0.05 were considered significant. The compliance to consumption of Wawamum was calculated by considering the number of days Wawamum was reported to have been consumed divided by the number of days of received and multiplied by a 100. Data was double entered in Visual FoxPro database. All analysis was performed using STATA version 15.

### Ethical considerations

Written informed consent was obtained from parents or caregivers at the time of child enrolment in the study. The consent forms provided information on the study objectives, anthropometric measurements, and confidentiality. The Ethics Review Committee (ERC) of Aga Khan University granted approval for the study on January 31, 2014. The study was registered on ClinicalTrials.gov with registration number NCT02422953 on April 15, 2015. However, enrollment of study participants started before final registration for organizational reasons but no changes to the protocol were made after this time point. The authors confirm that all ongoing and related trials for this intervention are registered.

## Results

### Baseline characteristics of children 6–18 months

A total of 870 children 6–18 months of age (419 in intervention; 451 in control) were enrolled in the study. Gender distribution was comparable in both groups. The mean age of children in the control (11.1 ± 4.2 months) and intervention groups (11.3 ± 4.1 months) was similar. Baseline stunting, wasting and anaemia rates were comparable in both control and intervention groups but the rate of underweight among children was higher in the control group (50.9%) as compared to the intervention group (40.1%) at enrolment. Access to improved drinking water, household food consumption score and household hunger score were comparable in both control and intervention groups [Table 2].

**Table 2:** Baseline characteristics.

Child characteristics	Control N = 451	Intervention N = 419
**Gender**		
Male	226 (50.1%)	214 (51.1%)
Female	225 (49.9%)	205 (48.9%)
Age (months)	11.1 ± 4.2	11.3 ± 4.1
**Malnutrition status**		
Stunting (LAZ < -2SD)	208 (47.8%)	183 (44.1%)
Wasting (WLZ < -2SD)	106 (23.8%)	85 (20.4%)
Underweight (WAZ < -2SD)	221 (50.9%)	167 (40.1%)
**Hemoglobin level**		
Normal (≥11gm/dl)	50 (11.1%)	50 (12.0%)
Anaemia (<11gm/dl)	400 (88.9%)	367 (88.0%)
Moderate Anaemia (≥7 & <11 gm/dl)	360 (80.0%)	337 (80.8%)
Severe Anaemia (<7gm/dl)	40 (8.9%)	30 (7.2%)
**Household characteristics**		
Improved water	396 (87.8%)	363 (86.6%)
**Food Consumption Score (FSC)**		
Poor (1–28)	3 (0.7%)	4 (1.0%)
Borderline (28.1–42)	2 (0.4%)	5 (1.2%)
Acceptable (>42)	446 (98.9%)	410 (97.9%)
**Household Hunger Scale (HHS)**		
None or light hunger (0–1 score)	375 (83.1%)	367 (87.6%)
Moderate hunger (2–3 scores)	72 (16.0%)	49 (11.7%)
Severe hunger (4–6 scores)	4 (0.9%)	3 (0.7%)

**Fig 1:**
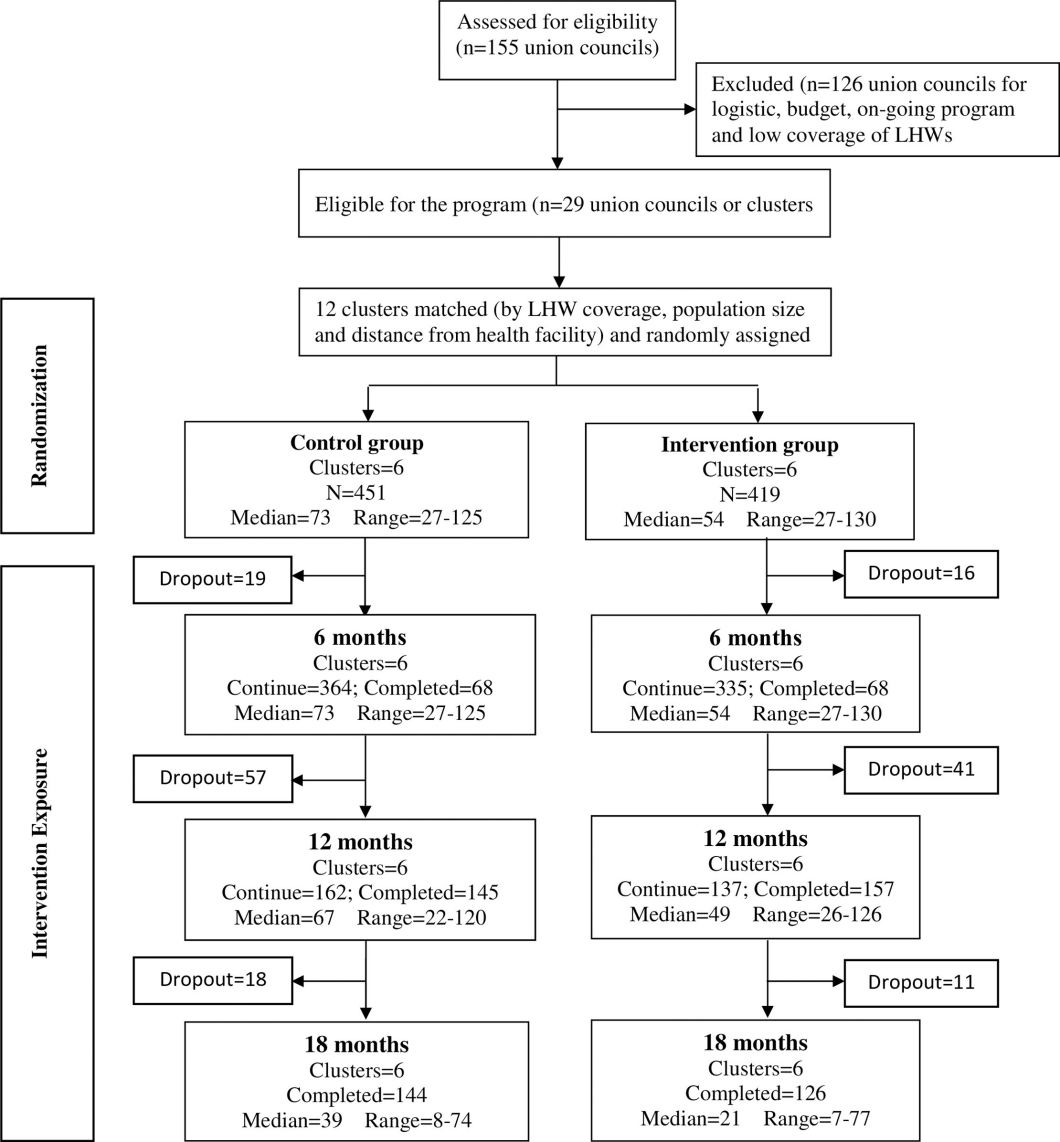
Consort flow diagram of trial.

Of the children enrolled in the study, approximately 5% (5.1% in control and 4.1% in intervention groups) were lost to follow-up. There was one death in the intervention group and four deaths in the control group. A total of 428 (94.9%) children in the control group and 402 (95.9%) children in the intervention group completed all their follow-up visits by 24 months of age or had follow-up assessments for at least six months [Fig 1].

### Compliance of Wawamum in children 6–23 months

Table 3 shows the compliance of Wawamum in children 6–23 months of age. The mean days observed during the study period were 378.9 ± 129.4 days. On average, each child consumed Wawamum for 266.9 ± 123.1 days. The mean compliance of Wawamum was 60.3 ± 19.8%. Overall, each child shared 40.5 ± 35.4 sachets of Wawamum with other family members during the study. Only 3.9% of children did not share any sachets of Wawamum with other family members while 43.1% of children shared 1–30 sachets, 29.7% shared 31–60 sachets, 14.5% shared 61–90 sachets and 8.8% shared > 90 sachets during the study.

**Table 3:** Compliance of Wawamum in children 6–23 months.

Indicators	N = 419
Days Observed, Mean (SD)	378.9 (129.4)
Days received, Mean (SD)	266.9 (123.1)
Days consumed, Mean (SD)	226.4 (101.2)
Percent compliance of Wawamum (days consumed/days observed*100), Mean (SD)	60.3 (19.8)
Number of sachets shared with other family members (Mean (SD)	40.5 (35.4)
Distribution of sachets shared with other family members, Count (%)	
No sharing	16 (3.9%)
1–30 sachets	176 (43.1%)
31–60 sachets	121 (29.7%)
61–90 sachets	59 (14.5%)
> 90 Sachets	36 (8.8%)

### Impact on nutritional status in children 6–23 months of age during the study period

Table 4 shows the impact of Wawamum on the nutritional status of children 6–23 months of age. Overall, there was a significant reduction in the risk of stunting (RR = 0.91, 95% CI; 0.88–0.94, p<0.001) and wasting (RR = 0.78, 95% CI; 0.67–0.92, p = 0.004) in the intervention group in comparison to the control group. No evidence was observed on reduction in risk of underweight (RR = 0.94, 95% CI; 0.85–1.04, p = 0.235) in the intervention group compared to control group.

**Table 4:** Impact on nutritional status in children 6–23 months during the study period.

Nutritional status	Age at exposure to LNS	Control*	Intervention*	^1^^,^^2^RR (95% CI)	p-value
		%	%		
Stunting	6–12 months	55.7	46.4	0.83 (0.81–0.86)	<0.001
(LAZ <-2SD)	13–18 months	60.5	54.9	0.90 (0.83–0.97)	0.008
(n = 850)	Overall (6–23 months)	60.5	54.9	0.91 (0.88–0.94)	<0.001
Wasting	6–12 months	22.5	19.3	0.86 (0.72–1.02)	0.088
(WLZ <-2SD)	13–18 months	23.1	18.2	0.79 (0.63–0.98)	0.035
(n = 862)	Overall (6–23 months)	20.5	16	0.78 (0.67–0.92)	0.004
Underweight	6–12 months	45.9	45.8	1.00 (0.84–1.18)	0.976
(WAZ <-2SD)	13–18 months	46.8	42.8	0.91 (0.81–1.03)	0.136
(n = 850)	Overall (6–23 months)	48.7	45.8	0.94 (0.85–1.04)	0.235

*Adjusted proportion by arm

1. Risk ratios and corresponding 95% confidence intervals and P-values obtained from generalized linear model using a log link and binomial distribution. When the log‐binomial model did not converge (in the models for stunting), a Poisson distribution was used.

2. All models adjusted for clustering and controlled for time on intervention and respective baseline nutritional status.

The subgroup analysis by age showed the intervention effect is significant in reduction of stunting in the younger aged children of 6–12 month (RR = 0.83, 95% CI; 0.81–0.86, p = <0.001) and the older aged infants 13–18 months (RR = 0.90, 95% CI; 0.83–0.97, p = 0.008).

### Anaemia status in children at 24 months of age

In total, 320 children in the control group and 322 children in the intervention group were assessed for their anaemia status at 24 months of age [Table 5]. There was a statistically significant risk reduction of anaemia in the intervention group compared to the control group (RR = 0.97, 95% CI; 0.94–0.99, p = 0.042). A highly significant risk reduction in severe anaemia was observed in the intervention group compared to the control group (RR = 0.45, 95% CI; 0.29–0.70, p = <0.001).

**Table 5:** Anaemia status in children at 24 months of age.

Anaemia status	Control n = 320	Intervention n = 322	*RR (95% CI)	p-value
	%	%		
Anaemia (<11gm/dl)	93.9	91.2	0.97 (0.94–0.99)	0.042
Severe Anaemia (<7gm/dl)	17.5	7.8	0.45 (0.29–0.70)	<0.001

*Risk ratios were estimated using generalized linear model with log (link) to account for cluster randomization and baseline status.

### Impact on diarrhoea and ARI in children 6–23 months during the study period

Overall, the prevalence of caregiver-reported diarrhoea during past two weeks during the study period among children 6–23 months of age was 51.2% in the intervention group and 49.0% in the control group, after adjusting for clustering and baseline morbidity (PR 0.84, 95% CI 0.60–1.17, *p* = 0. 295). Similarly, no difference in the prevalence of acute respiratory infection (ARI) among children 6–23 months of age was noted as 27.1% in the intervention group and 28.7% in the control group, after adjusting for clustering and baseline morbidity (PR 0.60, 95% CI 0. 31–1.15, *p* = 0.123) (Table 6).

**Table 6:** Impact on diarrhea and ARI in children 6–23 months during the study period.

Morbidities	Control (n = 4238)	Intervention (n = 3622)	^1^RR (95% CI)	p-value	^2^RR (95% CI)	p-value
Diarrhoea	2207 (49.0%)	1855 (51.2%)	0.84 (0.56–1.25)	0.387	0.84 (0.60–1.17)	0.295
ARI	1218 (28.7%)	982 (27.1%)	0.70 (0.31–1.57)	0.387	0.60 (0.31–1.15)	0.123

*n =* Number of monthly visits

1. Risk Ratio estimated using generalized linear Poisson regression model with link (identity) to adjusted for clustering

2. Risk Ratio estimated using generalized linear Poisson regression model with link (identity) to adjusted for clustering and respective baseline morbidity

## Discussions

Our study demonstrates that the provision of a lipid-based nutritious supplementation to children during 6–23 months of age was effective in reducing the risk of stunting, wasting and anaemia. We found a significant reduction in the risk of stunting (9%) and wasting (22%) in children aged 6–23 months receiving LNS versus children receiving no supplementation. There was non-significant impact also observed in the prevalence of underweight (6%) in the intervention group compared to the control group. Children in the intervention group were found to have a 3% and 55% decrease risk of anaemia and severe anaemia, respectively. Based on age at exposure to the LNS, we observed potentially greater impact of Wawamum on reduction in the risk of stunting who received intervention for longer period as compare to children for less duration of age (RR = 0.83, 95% CI; 0.81–0.86, p = <0.001) as (RR = 0.90, 95% CI; 0.83–0.97, p = 0.008).

In a recent study in Ghana, LNS-SQ was provided to mothers during pregnancy and lactation and to children during infancy (6–23 months); iron-folic acid (IFA) tablets were provided to women during pregnancy, and multiple micronutrient supplements (MMS) were provided during pregnancy and lactation [32]. The study found a significant lower prevalence of stunting among the group receiving LNS (8.9%) when compared to the IFA group (15.1%) and MNP group (11.5%; overall p = 0.045). However, unlike our study, supplementation was provided during the 1,000-day window in Ghana. Evidence from earlier studies in China [33], Haiti [18] and Niger [34] have shown that nutrition-based supplementation may reduce the prevalence of stunting in children. When a locally produced soybean-based supplement (Ying Yang Bao) was introduced to children 6–18 months of age in vulnerable areas of China, the prevalence of stunting, wasting and underweight decreased from 8.9%, 3.5% and 4.5% to 5.0%, 1.7% and 3.3%, respectively [33]. Overall, the growth and development status significantly improved after introducing Ying Yang Bao in children under two years of age.

We found a slight but significant reduction in anemia prevalence (91% vs 94%) and highly significant reduction in severe anemia (7.8% vs 17.5%) in the intervention children compared with control. The reduction in the prevalence of overall anemia in our study is lower than the studies conducted in China [33], Ghana [34] Burkina Faso [35] and Bangladesh [36]. This slight reduction in the prevalence of anemia may be due to low compliance of LNS-MQ as compared with these studies. However, 91% of children in intervention group were still anaemic at the end of the intervention, despite the daily provision of LNS-MQ during 6–23 months of age. Therefore, additional or long term interventions would be needed to reduce the high anemia burden in the study area.

In Haiti, there is an extensive history of complementary food supplementation among vulnerable populations. When provided for a period of six months, small quantities of daily LNS supplementation (20g/day) significantly increased height-for-age z-score (0.13 ± 0.05) and weight-for-age z-score (0.12 ± 0.02; p<0.001) as compared to children receiving no LNS in Haiti [18]. Furthermore, regardless of the quantity of LNS provided–large quantity of 92 grams and medium quantity of 46 grams–a similar reduction in severe wasting (RR 0.97; 95% CI 0.67–1.40; p = 0.88), moderate wasting (RR 1.20; 95% CI 0.76–1.19; p-0.67), severe stunting (RR 0.94; 95% CI 0.70–1.26; p = 0.69) and moderate stunting (RR 0.95; 95% CI 0.76–1.19; p = 0.67) was found in children 6–23 months of age in Niger [37]. The above-mentioned studies confirm our results, where children who are exposed to nutrition-based supplementation during the period of greatest nutritional vulnerability (the 1,000-day window) benefit more than children who were not exposed to the nutrition-based supplementation.

Our study has many strengths, including the use of existing government health system to deliver the intervention through LHW programme, cluster-randomized controlled study design, low rate of loss to follow-up and consistent data collection on a monthly basis. In addition, we conducted a 5% validation of the data collected through a separate verification team to ensure data quality. However, there are a few limitations. First, children were recruited between 6 months of age to 18 months of age. This recruitment process led to a large difference in exposure to the interventions among children (ranging from 6 months to 18 months of exposure). This may have reduced the impact of Wawamum on the nutritional status and anaemia levels among children. Second, sharing of Wawamum with other family members may have also reduced the impact on the nutritional status of children.

## Conclusions

A significant reduction in risk was observed in stunting, wasting and anaemia among children 6–23 months of age. Our study demonstrated the plausibility of achieving nutrition goals in the short-term and medium-term with nutrition-based supplementation delivered through the primary healthcare system. There is a need for a programmatic scale up of supplementation delivered through the existing LHW programme or other delivery systems, preferentially targeting those most in need (i.e. largest nutrient intake gap), which can be replicated in different areas of the country.

## Supporting information

S1 File (DOCX)

S2 File (DOCX)

S1 Checklist CONSORT 2010 checklist of information to include when reporting a randomised trial*. (DOC)

S1 Data set (ZIP)
