# Supplementary material for: Effect of lipid-based nutrient supplement—Medium quantity on reduction of stunting in children 6-23 months of age in Sindh, Pakistan: A cluster randomized controlled trial
Source: PLoS One. 2020 Aug 13;15(8):e0237210. doi: 10.1371/journal.pone.0237210 (PMC7425934; doi:10.1371/journal.pone.0237210)
Supplement: S1 File — (DOCX) [file pone.0237210.s001.docx]

Collaborators

Women and Child Health Division, Aga Khan University

World Food Program

Department of Health, Government of Sindh

Proposal

Impact evaluation of effectiveness of food based interventions to prevent stunting among 6-23 months children in Thatta & Sajawal Districts, Sindh Province Pakistan

Aga Khan University, Pakistan

**Project Core Team**

**Principal Investigator**

**Dr. Sajid Bashir Soofi,**

Associate Professor

Aga Khan University, Pakistan

**Co-Principal Investigator**

**Dr. Shehla Zaidi,**

Associate Professor

Aga Khan University, Pakistan

**Co-Investigators**

**Dr. Shabina Ariff,**

Assistant Professor

Department of Pediatrics & Child Health, Aga Khan University, Pakistan

**Dr. Muhammad Atif Habib,**

Senior Instructor,

Department of Pediatrics & Child Health, Aga Khan University, Pakistan

**Dr. Dureshawar Khan,**

Nutrition Focal Person, Government of Sindh

Chief Technical Advisor

**Dr. Khizar Tauseef Ahmed Ashraf**

Nutrition Programme Officer, World Food Programme

**Ms. Mona Shaikh**

Nutrition Programme Officer, World Food Programme

**Project Supervisor**

**Mr. Gul Nawaz Khan,**

Senior Social Scientist

Department of Pediatrics & Child Health, Aga Khan University, Pakistan

**Technical Advisors**

**Prof Zulfiqar Ahmed Bhutta**

Founding Director Center of Excellence in Women and Child Health,

Aga Khan University, Karachi, Pakistan

Co-Director, Center for Global Child Health, Hospital for Sick Children, Toronto, Canada

**Ms Angela Cespedes,**

Head of Nutrition, World Food Program, Pakistan

**Preamble**

Widespread food insecurity and malnutrition are largely the main impairing factors for human capital development in the Pakistan. Rates of chronic malnutrition are very high, and acute malnutrition is critical: 44% of children under five are stunted and nationwide global acute malnutrition (GAM) rates amongst children under five exceed the WHO critical threshold of 15%. Nutritional status trends also show a deteriorating situation since 1994, when stunting rates were at 36%.

Recurrent crises in recent years such as the devastating 2010 floods have contributed to a sharp decline in food security, despite sufficient national food production of staples. Currently, 104 million people, or 58% of the population, are estimated to be food insecure. National nutrition survey 2011 shows that stunting under-five children is significantly higher in food insecure households [1].

Children under two are consuming less than half of their daily energy requirements (560 kcal) and lower than recommended levels of micronutrients (one-third of RNI for Iron and one-half for Zinc). Overall, less than 4% of children were receiving an acceptable, diverse diet [2]. High under-five child morbidity is also noted- 22% of the children had ARI on the day of the survey and 12% had diarrhea, while another 22% had diarrhea in the previous two weeks [1].

There is not only the overall issue of appropriate child care practices but also of food diversity and nutrient quality. The widely spread food insecurity is attributable mainly to lack of access. Nearly 27% of the population is living in extreme poverty while nearly half are estimated to be suffering from multi-dimensional poverty and intensity of deprivations.

Food security and nutrition situation in Sindh province is even more dramatic. According to the National Nutrition Survey, 2011, GAM prevalence exceeds 18% while anemia levels amongst children under five reached the alarming level of 72.5% [1]. With this high level of poverty and food insecurity, a food based approach to prevent stunting, together with non- food based approaches is needed.

**WFP Response and Stunting Prevention Project**

The World Food Programme (WFP) is the [food assistance](http://en.wikipedia.org/wiki/Food_aid) branch of the [United Nations](http://en.wikipedia.org/wiki/United_Nations), and it is the world's largest [humanitarian](http://en.wikipedia.org/wiki/Humanitarian) organization addressing [hunger](http://en.wikipedia.org/wiki/Hunger). WFP provides food, on average, to 90 million people per year, 58 million of whom are children. WFP strives to eradicate [hunger](http://en.wikipedia.org/wiki/Hunger) and [malnutrition](http://en.wikipedia.org/wiki/Malnutrition), with the ultimate goal in mind of eliminating the need for food aid itself World Food Programme, Pakistan has started a three years PRRO 200250 (Protracted, Relief and Recovery Operation) 2013-2015, with a strong focus on nutrition. The main interventions are continuation of CMAM (Community management of acute malnutrition) in the selected 36 districts of the country. In addition, a pilot food based Stunting prevention intervention is to be initiated in Thatta district, Sindh Province [2].

Thatta was selected considering that is one of the 19 districts identified by the Sindh government to implement their Strategic Nutrition Response Plan to address high prevalence of acute malnutrition in children and pregnant and lactating mothers. In addition Thatta is one of the districts with very high food insecurity levels according to the food insecurity ranking conducted in 2009 and also with very high proportion of undernourished population (below 2100 Kcal) identified by WFP/FAO schlock modeling exercise in 2012.

Further Since 1999, Thatta district has experienced six disasters a cyclone in 1999, drought in 2000, earthquake in 2001, drought and floods in 2003, monsoon floods in 2010 and torrential rains in 2011. The 2005 socioeconomic study conducted by The World Bank and Government of Sindh in 2 coastal districts including Thatta found that 1.2 million people, or 175,000-200,000 households, living in the coastal sub-districts (Taluka) of the 2 districts are the poorest of the poor in Pakistan.

As mentioned, stunting is one of the major nutrition problems in Pakistan, affecting nearly half of the children under five years of age. As a manifestation of chronic under nutrition, stunting has been linked to multiple adverse health outcomes that extend into adult life and the future generations, being the most important the reduced physical and cognitive capacity. It has serious implications for educational achievement and future earning capacity of individuals translating into GDP losses for countries. Estimates published in the 1990s indicate that the losses to GDP for Pakistan from various components of under nutrition can be as high as 3 percent of national income [2].

As such prevention of stunting should be one of the priorities to ensure economic and social development. There is existent evidence that points to the need to intervene during gestation and the first two years of life to prevent child under nutrition and its consequences. It suggests that investments in interventions during this window of opportunity are likely to have the greatest benefits.

In Pakistan, high rates of stunting are attributable to multiple factors compounded by an environment of shocks and deteriorating food security situation. While the CMAM initiative, stimulated by the floods and their media attention as a lifesaving intervention, created a sense of achievement in the country's health sector, the NNS findings of increasing chronic malnutrition has generated a broad recognition in the government and within the donor community that a preventive approach was also needed.

Although a number of nutrition approaches and actions have been implemented in the country at various times, there hasn't been a comprehensive approach to prevent under nutrition in general, and stunting specifically. The project aims to contribute towards the evidence base for food based interventions for the prevention of stunting embedded in existing and proposed IYCF provincial plans.

**Objectives of WFP Stunting Prevention Project**

The overall aim of the project is to utilize the window of opportunity (1000 days from conception to 2 years) for addressing stunting in children under 5 with following specific objectives of the project:

- To reduce stunting in children 6-23 months using a locally produced LNS^^[[1]](#footnote-1)^^ type (Wawamum^^[[2]](#footnote-2)^^) and promoting appropriate IYCF practices (including promotion of exclusive breast feeding until 6 months).
- To reduce micronutrient deficiencies in children 24-59 months using multi micronutrient powders, MNP^^[[3]](#footnote-3)^,^, along with appropriate behavior change communication.
- To improve maternal/PLW nutritional status and to have an impact on the birth outcome (reduce low birth weight),
- To study effectiveness of food based approach to address stunting among children under-five years

The project will operate in 29 Union Councils (UCs) in Thatta and Sajawal districts, selected on the basis of LHW´s presence, since they are the main implementing partners. The design considers the supplementation of specialized nutritious food to all eligible children and mothers. The project will also support the implementation of existing IYCF programme, complementing where needed with an appropriate food based intervention to assist families in getting the 'right food at the right time'. In fact the project rationale considers food/nutrient supplements as a vehicle that adds value to the effect of the other longer term behavior change and preventive health interventions.

Although there is evidence available on the effectiveness of food/nutrient based approaches, in prevention of stunting from various studies being conducted in developing countries, but, considering the local factors and wide spread macro and micronutrient deficiencies in Pakistan there is a need for operational research of such interventions to document the impact and comparative advantage over other interventions, including the optimal length of supplementation. Many efficacy studies were undertaken under controlled conditions such as randomized trials, but the results of these studies cannot be directly extrapolated to programmes operating under field conditions, frequently with low capacity and multiple limiting factors [3].

Therefore there is a growing need for stronger evidence base on the effectiveness of such interventions under “real” operational conditions. In this line, WFP project in collaboration with Government of Sindh, Health Department, through its research component proposes to strengthen the evidence on preventive food/nutrient based approaches for reducing stunting and developing viable programmes while also investing in national capacity development on nutrition.

## WFP Intervention Modalities and Targeting Criteria:

To achieve its purpose and objectives the project design includes core interventions such as complementary feeding using food/nutrient based supplements along with behavior change communication, complemented with other non-food interventions. Using a preventative (blanket) approach, three types of food/nutrient supplements are considered as part of the interventions:

- Distribution of locally produced LNS (Wawamum) to children from 6 – 23 months, this group will receive a daily ration of 50 g of Wawamum that covers the total RDA for most of the micronutrients and approximate one fourth of the daily energy requirements.
- Distribution of micronutrient powders to children 24-59 months: This group will receive a sachet of MNP that covers the total RDA for 15 micronutrients in alternate days.
- Distribution of “Super cereal” fortified blended food (Wheat Soya Blend., WSB) to PLW: A monthly ration of 5 Kg of WSB will be given to this group every month during pregnancy and for six months after giving birth

Besides the provision of food/nutrient supplements for children and PLW and the education on product use and benefits, through the LHWs and in coordination with the health facilities and other partners, the programme will support other interventions related to infant and young child feeding promotion, starting with the newborn´s care (early initiation and exclusive breastfeeding, sustained breastfeeding, complementary feeding and hygiene practices) and maternal nutrition education. The contents for the nutrition education will be the same as those developed and adapted to the country by the Health sector and UNICEF. The nutrition messages will be delivered by the LHW through group sessions, demonstrations and household visits. It is planned to conduct a formative research to tailor the key messages to the local context of Thatta district and also to identify more effective communication methods.

**Complementary interventions**

These are interventions not directly supported by the project but planned or already in place by other stakeholders and also by the LHW programme itself. These actions address other crucial factors to reduce stunting rates such as preventive health care. The project will be linked to these activities through an inter-institutional coordination platform, exclusively constituted for Thatta, convening all actors involved on nutrition.

Complementary interventions include the following:

**Pregnant and lactating women:**

- Promotion of antenatal and post-natal care
- Immunization against tetanus
- Safe delivery
- Family planning counseling
- Provision of iron and folic acid supplements
- Prevention and treatment of common diseases, including malaria prevention
- Timely identification of dangerous signs during pregnancy and referral to health centers

**Children 0-59 months**

- Immunization
- Treatment of common diseases, including malaria prevention
- Treatment of acutely malnourished children: inpatient and outpatient care

The above activities are being delivered by the health sector and the LHWs. In addition MERLIN through its EU funded project will reinforce all the behavior change, community outreach and CMAM activities in most of the UCs in the district.

## Study Site and eligibility criteria

According to the implementation plan of WFP, the project will be operating in 29 Union Councils (UCs) in Thatta and Sajawal districts. The UCs were selected on the basis of LHW´s presence, since they are the main implementing partners. The project aims to reach children 6-23 months, children 24-59 months and PLW.

Inclusion criteria for children and mothers are defined as follows:

**Children 6-23 months:** All eligible children between 6-23 months in the catchment area will be enrolled for food supplements, independent of nutritional status and age. This group will receive Wawamum during the programme period.

**Children 24-59 months:** All eligible children between 24-59 months in the catchment area will be enrolled for food supplements, independent of nutritional status and age. This group will receive MNPs during the programme period.

**Pregnant and lactating women:** All pregnant women will be enrolled in the project, regardless of the month of pregnancy and nutrition status. The programme will try as much as possible to engage women at earliest stage of the pregnancy. Similarly all lactating women in the first six months after giving birth will be engaged, covering the exclusive breastfeeding period. It is also being discussed whether it will be important a screening to identify overweight or obese women and what would be the approach in such cases.

**Project Delivery Mechanism**

The project will be implemented in collaboration and with the support of Government of Sindh Health Department, specifically the Nutrition cell. The delivery strategy is based on the existing and functioning community-based platform which is the national programme of LHWs. The provincial coordinator of LHW´s programme of Sindh will act as the Project Director from the Government side. However for smooth implementation and for day to day correspondence a focal person will be nominated by PPIU Sindh of LHWs programme. In total the project will be involved more than 500 LHWs in the project area. Other implementing partners are UNICEF, WHO, PPHI, MERLIN, PDHC and Academia. All stakeholders will convene in a coordination platform established for Thatta district and will meet once a month.

**Beneficiary Registration**

All eligible children and women in the LHW´s catchment area will be registered before the first distribution by the LHWs as part of their routine activities. Thereafter, all newly pregnant women, lactating mothers and children 6-23 and 24-59 will be registered also as part of the normal LHWs duties.

After registration, the beneficiaries will receive a "programme ration card" that contains the registration number of the beneficiary (PLW/child) which will be used to receive the appropriate supplement.

At the moment of enrollment children´s height, weight and MUAC will be measured. Weight will be also measured on a monthly basis, while height will be measure at discharge time.

**Transportation of Commodities**

WFP will facilitate the transportation of the food supplements up to District level and storage in a warehouse managed by WFP together with the district level team of the LHWs programme. Distribution to the health facilities and subsequently to the LHWs will be arranged by the LHWs programme. The LHWs will be then responsible to transport the commodities to their health house in the villages and distribute to the final beneficiaries. Cash support to cover the transport cost from the health facility to the health house will be given to each LHW. A robust supervision and control of product inventories at each stage is necessary to guarantee the timely distribution to beneficiaries, and to prevent any damage or diversion of the products. In selected union councils for the CRT the intervention delivery will be monitored by the third party which is AKU in this case.

**Training of the LHWs**

The LHWs provincial and district level staff, health sector staff involved in the project and the LHWs and supervisors will attend a two days training before starting the distribution of food supplements. A cascade training approach will be followed, first training Master trainers for each health facility, followed by the LHWs training. Main topics for the trainings are: the benefits and use of the products, the delivery strategy and reporting. A brief refreshment session on IYCF and maternal nutrition will be also included (it is assumed that LHWs are already trained in these topics).

**Supplementary products delivery and education on its benefits and use**

Beneficiary PLW and children´s mothers or caretakers will receive the food/nutrient supplements in the LHW health house on a monthly basis. When beneficiaries and caretakers receive the product, they will also receive education around product benefits and use. There are three options for providing product education short education sessions at delivery points; messaging & discussions via community care groups for mothers; additional personal counseling at household visits. According to the LHWs programme routine services, LHWs has to visit all the households in their catchment area at least once a month.

**Delivery of IYCF and PLW nutrition education**

Messages will be delivered by the LHWs at the household visits. During the monthly household visit, the LHWs will weight all 6-23 months children and register the weight in the growth monitoring chart. Nutrition counseling will be then provided according to child´s age and direction of the weight monitoring chart. More attention will be given to those children that lost weight or not gained weight; inquiring on the possible causes and providing advice on corrective measures related to improved child feeding and care practices.

Other complementary activities such all preventive health is being implemented by other stakeholders, as mentioned above.

**Food distribution and beneficiary monitoring**

WFP will be responsible for supervising/coordinating the overall project monitoring which includes timely data collection and analysis, and reporting. WFP monitoring staff devoted to the project will include a nutrition officer based in WFP Karachi, one or two field monitors based in the district Thatta.

The monitoring of beneficiary registration and food delivery will be carried out by the LHWs programme staff and supervised by the WFP field monitors (through spot-checks, post-distribution monitoring interviews and data analysis).

Information on number of beneficiaries and quantities of supplements distributed will be submitted on a monthly basis by each LHW to their supervisor (LHS). The LHS will compile manually the information submitted and send it to the district coordination for compilation and submission to WFP and provincial level coordinators. WFP will support the District coordination to develop and implement a distribution control and inventory management system.

Every month WFP field monitors will conduct a post-distribution interview to a randomly selected sample of beneficiaries. This will focus on the satisfaction of the beneficiaries with the project service delivery and the use of the products within the household.

The whole process of food distribution will be monitored by the third party as well which is AKU in this case. The surveillance team from AKU will collect routine data on compliance and observation of available and used empty packets/sachets.

**Rationale of an Independent Impact Evaluation of Stunting Prevention Project by Aga Khan University**:

An impact evaluation assesses changes in the well-being of individuals that can be attributed to a particular project, program or policy. It is aimed at providing feedback to help improve the effectiveness of programs and policies. Impact evaluations are decision-making tools for policymakers that promote accountability to the public [4]. Although there are other types of program assessments, including organizational reviews and process monitoring, but these do not estimate the magnitude of effects and assign causation. Such a causal analysis is essential for understanding the relative role of alternative interventions in reducing poverty. [5]

While the project is planned as an extensive exercise with its own process evaluation and information pathway, it is critical that the true impact and benefit of this program is evaluated objectively. This must be achieved as an independent process in parallel with the primary activity in order to have the requisite credibility and the basis on which its future integration within provincial and national health programs can be decided. Such objective and in-depth evaluation for effectiveness is therefore an essential part of the program and will be needed to justify future continued investments in this intervention.

In general, a credible third-party observation is considered less biased and more inclusive of multiple viewpoints. These are valuable qualities that can help promote the acceptance and use of evaluation results. In general, internal project staff is concerned with gathering and using formative information for developing and improving implementation of specific strategies. Being able to tie various types of evaluation results (fidelity, process and summative) together is essential for evaluating the overall progress and outcomes of a project, and is best completed by someone external to a project.

Therefore, Division of Women and Child Health, Aga Khan University proposes a research study for stronger evidence base on the effectiveness of preventive food/nutrient based interventions on reduction of stunting and developing viable programmes on nutrition under “real” operational conditions.

**Objectives of impact assessment of Stunting Prevention Project by AKU**

The broad objectives of the research component of the project are:

1. To assess the effectiveness of the food/nutrient -based interventions on reduction of stunting among children under two.
2. To assess the effectiveness of the food/nutrient-based interventions on reducing stunting and micronutrient deficiencies in children 6-59 months.
3. To assess the effectiveness of the food/nutrient-based interventions on reduction of wasting and anemia in pregnant and lactating women and an impact on the birth outcome (reduce low birth weight),
4. To assess the design and operational factors at the different stages of the programme cycle that may have affected the outcome.
5. To determine unintended consequences of the interventions, both positive and negative.
6. To determine cost-effectiveness.
7. To provide recommendations for improved programme design and evaluation.
8. To know the optimal length of preventive intervention.
9. To compare the use of specialized nutritious foods over other interventions, such as conditional cash transfers for stunting prevention.

**Key Questions:**

Key questions to be answered by the effectiveness assessment and operational research in order to inform the relevant stake holders on potentials and adjustments to this type of programme implementation are proposed as follows:

- Does the intervention have an effect on the nutrition outcome indicators (specifically stunting)? And why yes or why not?
- What would be the recommended length of feeding for infants with the nutritional supplement, i.e. 6-12 months, 6-18 months, or 6-23 months of age?
- What would be the recommended length of feeding for young children with the nutritional supplement, i.e. 24-30 months, 24-36 months, 24-48 months, or 24-59 months of age?
- What are the appropriate delivery mechanisms to reach the target populations, including the ultra-poor and hard to reach populations? What barriers exist to access these populations and what are possible solutions?
- What should be improved in the supply chain management to ensure timely delivery and maintain the nutritional quality of the products?

- What are the key drivers for behaviour change of target groups, within different income quantiles of the community to increase demand and use of specialized nutrition products?

**Methodology and Study Design:**

The effectiveness of the project will be measured in terms of the impact of the proposed interventions on the stunting and micronutrient deficiency prevalences in the target group (children and mothers). Given the conditions of project implementation, a quasi-experimental “double difference” design would be appropriate to assess the impact of the intervention. We propose to compare the intervention and non-intervention (control) groups before (first difference) and after the intervention (second difference).

Then, the operational implementation of the research comprises a baseline and end line surveys. In addition, a nested cluster randomized controlled trial will be implemented to track the evolution of key variables related to the quality of intervention delivery and intermediate nutrition outcomes. The control clusters will receive routine public and private health services available in the area. The overall research design is shown in Figure 1.

**Figure 1: Overall research design**


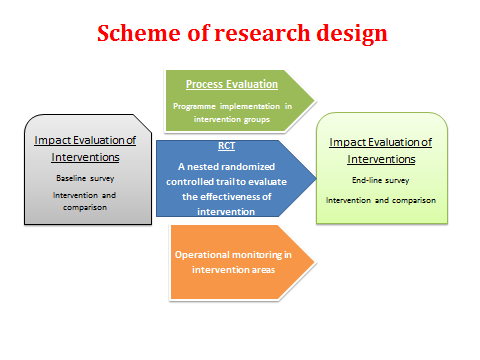


To ensure the robustness of this program a baseline and endline surveyswith a nested cluster randomized control trial to evaluate the programme in terms of nutrition and other core indicators is required. Therefore Division of Women & Child Health Aga Khan University proposes two separate cross sectional surveys which will be conducted in the target union councils of District Thatta-Sajawal to address the objectives of impact assessment of stunting prevention project. A baseline cross sectional survey will be conducted to provide benchmark indicators and frame work for the implementation of program. A structured questionnaire will be used to accomplish this information. At the end of the program an end line cross sectional survey will be conducted to assess the impact of the program, where the nutrition and other core indicators will be compared for pre and post intervention change.

**Study site**

This study will be conducted in 29 Union councils of districts Thatta and Sajawal of Sindh Province. The LHW catchment area of 29 targeted union councils of these districts will be in the intervention group, while remaining uncovered area of these union councils will be in control group. The UC wise details of LHWs, total population as well as covered and uncovered population are attached in annex I.

**Description of Proposed Activities:**

**A: Baseline and End Line surveys**

A cross sectional baseline and endline surveys will be conducted at household level for socio-economic status and nutrition related indicators. The baseline & end line surveys will be completed within three months. Following information will be collected from children under five years of age and pregnant or lactating women where available in selected households.

**Proposed Methodology for Baseline and End line Survey**

**1-Household size, socio-economic, household expenditure, hand washing, exposure to interventions, IYCF, food consumption & diet diversity, and morbidity information**

Household information will be captured from a pregnant woman or mother of under-five children. A structured questionnaire will be used to assess socio-economic status, family size, household expenditures, infant and young child feeding practices; exclusive breastfeeding and complementary feeding status. Additionally data on food intake, food consumption & diet diversity and food insecurities will be collected during baseline and endline surveys. Knowledge, attitudes and practices regarding micronutrients and hand washing practices will also be collected. The quantitative information will include precise energy, exposure to interventions, nutrient and food intakes estimates along with supplementation and fortification details. Apart from the above mentioned information, child’s health status, vaccination history and details of morbidity due to diarrhoea and pneumonia will also be collected in baseline and endline surveys.

**2- Anthropometric measurements, haemoglobin testing and physical examination.**

Height, weight and mid upper arm circumference (MUAC) measurements will be taken for all target population. Children’s ages will be determined from birth certificate, immunization card or other documentation, if available at the time of survey. If no such documentation is available, a local calendar will be used to determine age to the nearest month. Anthropometric indicators of length/height-for-age, weight-for-age and weight-for-length/height will be determined from above mentioned data. Physical examination will be performed of all targeted population to assess the signs of anaemia. In addition to the General examination of children and mother a spot haemoglobin test will also be done using a Hemo cue machine to assess the prevalence of anaemia.

**Sample size and design for Baseline and End line Surveys**

Clearly it would be impossible to conduct an in-depth evaluation of all population in the community based nutrition program. However, the assessment must be robust, scientifically valid and plausible within existing time frames. Therefore a representative sample size has been calculated separately for both baseline & endline surveys and embedded Randomized control trial. Details of both estimations are as under:

Below mentioned formula for sample size estimation for baseline and endline cross sectional surveys were used from MI & CDC manual [6] to calculate the sample size. Prevalence of stunting (49% Sindh) from NNS 2011, 80% power to detect 10% reduction in prevalence of stunting during the three-year implementation of the Stunting Prevention Programme with a significance level of 0.05 was considered for sample size estimation.


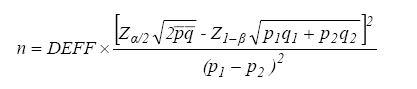


Assumptions:

- p1 is stunting rate at baseline without intervention at baseline survey =49%
- p2 is estimated stunting rate after intervention at end line survey
- DEFF : The estimated design effect = 1.5
- α : Level of significance (“alpha”), usually 0.05 or 5% (corresponds with 95% confidence interval)
- 1- β: Power, usually 0.8 (80%) Assumed 10% reduction in stunting

For estimating impact of the interventions each group will require 3200 participants. To account for dropouts and data errors, the sample size will increased by 10% giving a new total of 7040 study participants (3520 participants per group). Information will be collected from 4,200 households at baseline and at endline. The study participants from selected households will be pregnant women, lactating women and children under five years. Two stage cluster sampling technique will be used. LHW catchment areas of 29 UCs will be treated as a cluster for intervention group and control group.

All household with pregnant women, lactating mothers and children under five years of age will be eligible for baseline survey. LHW khandan register will be used to identify and select the eligible subjects in intervention clusters where available. A unique identification number will be assign to both children and mothers at the time of recruitment for the baseline survey. Social mapping approach (meeting with community elders, mothers, dais, community midwife, community/social workers etc.) will be used to identify the eligible subjects in control clusters. A list of eligible subjects will be prepared based on social mapping method and households with eligible subjects will be randomly selected for baseline survey.

Similarly all households with pregnant women, lactating mothers and children under five years of age at the time of endline will be eligible for endline cross sectional survey. Eligible subjects will be randomly selected from both intervention and control areas to see the overall impact of intervention on target population.

**Outcome Indicators for the survey**

| **Topics** | **Key variables** |
| --- | --- |
| Basic demographic characteristic of the household | Sex of the household head and children being assessed  Education level of the head of household and the spouse  Household size  Occupation of respondent and household head |
| Socio-economic characteristics of the household | Household wealth index  Type of the dwelling  Household residence status  Access to land and animals ownership |
| Household access to health services, water and sanitation | Distance to the nearest health facility  Main constraints to access health services  Presence of LHW  Main source of drinking water  Type of sanitation facility |
| Women´s care capacity | Women´s education level  Women´s reproductive history  Antenatal / postnatal care / immunization during pregnancy  Knowledge about vitamins , Anemia, iodized salt |
| Women’s physical examination and anthropometry | Physical examination for signs of anemia  Hemoglobin test results  Anthropometry |
| Household and children feeding practices | Prevalence of:   - exclusive breastfeeding - children ever breastfed - timely introduction of solid and semi-solids - early introduction of liquids - adequate dietary diversity - minimum meal frequency - minimum acceptable diet - consumption of iron-rich or fortified foods   Household food consumption and diet diversity  Consumption of iron-rich or fortified foods  Consumption of iron and folic acid supplements  Household coping strategies  Food insecurity |
| Children health status | Prevalence of reported illness (Fever, Diarrhea and ARI)in the past two weeks  Treatment type  Type of care provider  Immunization status |
| Child physical examination and anthropometry | Physical examination for signs of anemia  Hemoglobin test results  Anthropometry |
| Exposure to other interventions | Existence, type and coverage of other interventions related to food security, health and nutrition  Percentage of households, women and children already benefiting from interventions |

**Operational Survey Procedures**

**Development of Survey Protocol and Manuals:**

At the outset a detailed survey protocol will be developed in the first phase of survey. The protocol will cover both component of project.

**Development of Instruments:**

Data collection instruments will be developed in English and will include information on demographics; socioeconomic status; knowledge, attitudes and practices (KAP) and information related to the maternal and child health, nutrition status, food consumption & feeding practices and food insecurity. Similarly a recruitment form for the eligible children and PLW will also be developed. The project team will also develop the quarterly surveillance form through which the data will be collected during the quarterly surveillance rounds. The questionnaires will be translated into Sindhi and then back translated into English to assess whether the essence of the questions had been captured.

**Team Structure for Quantitative Component**

The project involves both major components which are data collection at household level, Anthropometric measurements and haemoglobin testing. Following is the team structure that has been proposed for the impact assessment study.


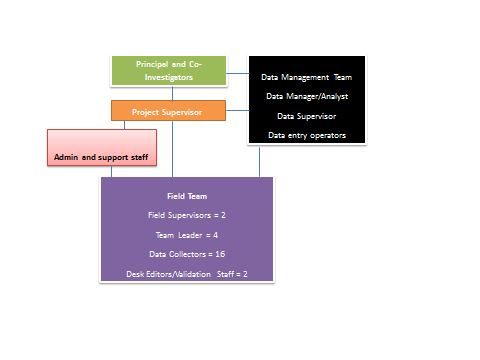


All project team members will be selected locally from Thatta and Sajawal. Female data collectors will collect the required information from the women and will conduct anthropometric measurements and Hb testing of the selected children and women. A male team leader will lead his team comprised of four female data collectors.

**Training**

Three trainings one for baseline, one for end line and one for the CRT will be done during the course of the project. Five days intensive training workshop will be organized for all local staff. This training workshop will be organized in four phases:

- One day combined session for all field staff (team leaders, data collectors and community nursing team) on background, objectives, methodology and timelines of the study.
- Two days session for data collectors on assessments of project and anthropometry
- One day joint mock field activity for the conceptual clarity on study procedures
- One day, feedback on the field visit and activities and finalization of teams

**Proposed Training scheme**

| **All participants** | **Team Leaders** | **Data collectors** |
| --- | --- | --- |
| General Introduction and Survey Methodology | Supervision skills  Quality assurance  Sampling methodology  Documentation  Reporting | Interviewing skills  Consent procedures  Q by Q Instrument review  Sampling Methodology  Operational Procedures  Field Procedures, Daily documentation /log sheets  Physical examination, Hemoglobin testing  Anthropometry  Field Practice |

The training agenda will cover the following points:

- Purpose of the project
- Roles and responsibilities
- Introduction to two stage cluster sampling technique and related issues
- Interviewing techniques
  - Selection of place for the interview
  - Consent taking and describing the process and the project
  - Attendance of others during the interviews / gaining community support
  - Probing/Rewording
  - Be prepared for any answer
  - Listen to the respondents
  - Answering respondents objections
  - Stress benefits of the survey
- Definition and technical aspects of different methodologies and terminologies
- Selection of procedures and use of interview tools
- Explanation of the questionnaires, question by question practice through simulated interview
- Field practices (mock interviews)
  - Critique of field practice
- Administrative procedures
- Anthropometry technique
- Clinical examination for anemia +Hb
- How to handle refusals
- Field quality checks
- Questionnaire review for completeness
- Quality Control and Quality Assurance
- Confidentiality and ethical issues

**Field Testing:**

Field testing will be performed on all aspects of the project. The questionnaire will be translated into the local language and tested. Field testing is useful to estimate the amount of time it will take to complete the interview and to identify any potential problems with the survey instruments and protocol. The field testing will be undertaken in conditions similar to the field within districts Thatta and Sajawal to simulate the actual data collection. Questionnaire validation and other community-level training activities will take place in areas different from those selected for the project. Following the field test, all project teams should convene and discuss the experience and modifications can be made, if necessary, to the protocol and data collection tools.

**Liaison with the Local Partner:**

Consultative meetings will be done with all relevant stake holders before the finalization of project protocol and methodology.

**Data Collection & Monitoring**

The data will be collected by trained female data collectors at household level; they will be allocated a set of households on daily basis. The whole activity of the data collection will be supervised and monitored by a team leader and field supervisor who will be trained to supervise this task. Morning meeting will be being conducted by the team leader/supervisor every day. An informed consent will be taken from the respondent in every household from where the data is collected. After field work data collection teams will return to the office for rechecking of forms before handing it over to concerned supervisor. The team leader will check the entire filled questionnaire for completeness before hand over to field supervisor. Field supervisor and desk editor will review the filled questionnaire for errors and inconsistencies. In case any errors and inconsistencies are identified the forms will be given back to the data collectors for correction from the field. After review and coding, all forms will be handover to DMU for data entry.

**Proposed Monitoring & Supervision Checks at Different Level:**

Effective supervision and monitoring is key to the success of any project. The project activities will be regularly monitored by various staff; the team leader will monitor the activities of every data collector. There will be several cross checking level during the assessments to ensure quality of data collected. Suggested checks during the survey are schematically presented in the figure below:

**At the end of each household**, the team leader should check if;

- Questionnaire headings such as name, date and household number are properly filled in
- All the questions has been answered
- Age has been correctly estimated and proper source is used (Birth certificate, local event calendar, vaccination card or any other official document verifying age)
- Weight and height has been properly entered
  - No rounding off of these variables.
  - Skip pattern has been respected

**Before leaving the area**, the team leader should check;

- The number of questionnaires corresponding to the number of households
- Any missing and absent subject should be checked
- Ensure all the absent subjects are visited again at the end of day

**At study office,** supervisors should check the selection of household, refusals, and check all questionnaires for completeness. In addition randomly select 5 % of households for revisits to cross-check the responses and the measurements.

**B. Impact Assessment of the Project:**

**Nested Cluster Randomized Controlled Trial**

During intervention delivery, a randomized controlled trial will also be conducted on a sub-set of targeted subjects for further robustness and to get the in-depth evidence about the impact of interventions on health and nutrition outcomes.

The Aga Khan University proposes an embedded Cluster Randomized Controlled Trial for more robust evaluation of the intervention being carried out by WFP. This CRT will be done at union council level to assess the acceptability, feasibility and effectiveness of food based interventions to prevent stunting among children in community settings. The project will utilize the window of opportunity (1000 days from conception to 2 years) for addressing stunting in children under-five years. Therefore project will recruit three types of cohorts in the study i.e. children 6-23 months, children 24-59 months and pregnant women to follow the newborns who delivered from recruited pregnant women.

**Methodology for Cluster Randomized Controlled Trial**

**Sample Size Estimation:**

A union council has been taken as the cluster for the estimation of sample size for RCT. The following three sample sizes were calculated to detect a range of differences in nutritional status for all study participants;

1. Pregnant and Lactating Women: To calculate sample size for PLW, we aimed to achieve a 25% difference in the prevalence of low birthweight. With a power of 0.80 and a statistical significance of 0.05, we calculated that a sample size of 160-170 per cluster would be sufficient for analyzing the reduction in low birthweight. A total of 2,000 PLW will be enrolled in the trial.
2. Children 6-23 months: Sample size for children aged 6-23 months was calculated to detect a 10% difference in risk reduction in stunting among the intervention group compared to the control group. A sample size of 70-75 children per cluster was estimated with a statistical significance of 0.05, power of 0.80, and an intra-cluster correlation of 0.02. A total of 900 children, 6-23 months old will be enrolled in the trial.
3. Children 24-59 months: To detect a 10% difference in risk reduction in stunting among the intervention group compared to the control group, a sample size of 90-95 children per cluster was drawn, with a statistical significance of 0.05, power of 0.80, and an intracluster correlation of 0.02. A total of 1100 children aged 24-59 months will be enrolled in the trial.

The total sample size will be taken from 12 union councils which will be randomly selected from the list of 29 union councils, 6 union councils for intervention group and 6 union councils for control group.

**Independent Data Collection System**

An independent data collection system will be established through trained research data collectors in addition to the baseline and endline assessments for the regular operational monitoring of project outcomes.

**Recruitment of potential participants:**

Children 6-23 and 24-59 months of age and Pregnant or lactating mothers will be potentially eligible cohorts for inclusion in the study and will be identified through the baseline survey being done in the targeted union councils. Pregnant women will be enrolled during first and second trimester of pregnancy, children 6-23 months cohort during 6-18 months and children 24-59 months cohort during 24-54 months of age for minimum exposure of six months. A detailed recruitment form will be filled from each study participant. Detailed physical & anthropometric examination and sport Hb testing will also be done at the time of recruitment.

**Monthly follow-up for compliance assessment:**

The project team will also conduct monthly follow ups of the recruited children and PLW for the assessment of compliance of the commodities. The compliance will be assessed via observation of used packets of commodities and parental recall.

**Quarterly Surveillance for monitoring of process and outcome indicators:**

The independent project team will conduct a quarterly (every three months) surveillance of the recruited children and PLW for the detailed anthropometric examination, compliance assessment, hemoglobin testing and programme related data at household level. This surveillance will enable to monitor the linear growth of children and its relationship with the intervention. Temporal trends of malnutrition will be the main outcome of this surveillance system. Over a period of one year intervention there will be four surveillance rounds.

**Collection of Blood Samples:**

About 3-cc blood samples will be collected from 500 children during 6-8 months of age and at 24 months of age from cohort of pregnant women and lactating mother’s group. The purpose of blood collection is to assess micronutrient deficiencies and impact of intervention in children. Children will be selected from both intervention and control areas after getting informed consents from their parents. This will enable us to compare improvements across intervention and control groups.

**Outcome Measures:**

**Primary Outcome Measure:**

The primary outcome will be reduction in risk of stunting in intervention group compared with control group.

**Secondary Outcome Measures:**

- Reduction in low birth weight in newborns
- Mean change in weight-for-height z-score (WHZ).
- Improvement in infant and young child feeding (IYCF) indicators.
- Linear growth velocity (HAZ increment/month).
- Mean hemoglobin concentration in children 6-23 months and 24-59 months of age.
- Prevalence of childhood anemia (Hb concentration<11g/dL) in children 6-23 months and 24-59 months of age.
- Mean hemoglobin concentration in mothers.
- Prevalence of maternal anemia (Hb concentration<12g/dL).
- Maternal BMI
- The change in BMI z-score over the intervention period

To calculate HAZ, WHZ and WAZ scores the 2006 WHO growth reference will be used. Hemocue machines will be used to measure Hb concentration.

**Reporting**

The project supervisor and data management unit will generate fortnightly indicator report for anthropometry and other process indicators. These reports will be shared with all stake holders.

**Plan of operations**

To ensure that various activities will be undertaken within the scheduled time for fieldwork, a comprehensive plan of operations will be planned as follows:

- Identification of resources
- Dialogue with the community elders / representatives
- Planning of field activities
- Questionnaire design and pre-testing
- Recruitment of field staff
- Training of field staff
- Mapping and selection of household
- Data collection & anthropometry measurements
- Data entry and cleaning
- Data analysis & reporting

**Quality Assurance**

To ensure proper implementation of study activities, the project supervisor will make spot checks and will give feedback to data collectors. In addition, 2-5% households will be re-interviewed within 24 hours of the original interview. For, objectivity, the staff involved in the monitoring will be totally independent of other project staff and will be specifically trained in interviewing and anthropometry assessments. Standard quality checks will be strictly enforced for anthropometric measurements.

**Data Management:**

**Editing, Coding and Data Transferring**

Data collection will be recorded on paper questionnaires. All interviews will be conducted in Sindhi. All collected data will be cross-checked by field supervisors after the data collection on a daily basis and will be transferred to the Data Management Unit prior to data entry, all forms will be checked for completeness and consistency. In case of inconsistency or missing responses, the editors will flag the errors/omissions and consult the data collectors for possible explanations.

**Data Entry and Data Quality Assurance**

All data will be double entered for purpose of sufficient accuracy. Data quality will be assured by performing dual and error checks simultaneously during entry.

**Software**

Visual Fox Pro will be used for designing of databases, data entry software and procedures for data quality assurance. Data entry screens will employ range and consistency checks and skips to minimize entry of erroneous data. Special arrangements will be made to enforce referential integrity of the database so that all data tables are related to each other without problem. Analysis of data will be done through SPSS version 18. The anthropometric data will be managed through the WHO Anthro software however for the day to day monitoring and quality assurance of data ENA SMART software will be used.

**Data Analysis**

For data analysis SPSS version 18 will be used and data will be analysed using univariate and multivariate methods. Statistical Analysis will be performed after the availability of clean and quality data. Each file will be converted from Fox Pro into SPSS files so that they could be read into SPSS for further analysis. Descriptive statistics for the subjects will be obtained and Pearson Chi square test will be used to establish association between categorical variables. Simple frequency tables will be generated to ascertain the information on Socio-economic and demographic data. Bivariate analysis will be done to establish association with the various factors and growth parameters such as stunting, wasting and underweight. Data analysis will be conducted based on the agreed dummy tables.

**C. OPERATIONAL DESIGN, UNINTENDED CONSEQUENECS & COST EFFECTIVENESS**

**Readiness for Food Based Intervention & Stakeholder Preferences on Design**

This section will aim at identifying key barriers and solutions for food supplementation to inform program design. It will be conducted at the time of baseline assessment. Specifically it will look into factors that may affect the overall operational research design, supply distribution chain and compliance of pregnant mothers and children.

*Acceptability:* Willingness for food supplementation program in the community will be explored across different stakeholders and need for advocacy/ communication in specific areas. Information will also be solicited to avoid contamination and cross over from Control to Intervention districts.

*Proposed Food Delivery Systems:* A number of aspects will be explored for proposed delivery systems and will include capacity, preferences and proposed measures for i) distribution outlets; minimization of supply breaks; adequacy of food storage; monitoring of food distribution; and minimization of pilferage and political capture. Also probed will be LHW presence on ground, and supporting factors that may be undertaken to enhance horizontal accountability of LHWs. The role of commercial sector in potential distribution and preparation of food will also be examined as well as the role of other district stakeholders in supporting the program in specific areas.

*End User Related Issues:* Client related issues will be examined in discussion with proposed users as well as local stakeholders. This will include product related factors such as demand for pre-prepared versus local foods; specific product preferences that may affect consumption. Decision making for use of prepared food in pregnancy and early childhood will also be probed in terms of cultural beliefs that may interfere with food use and identification of decision makers other than mothers who may influence use of food supplements. Barriers to IYCF will be explored including mothers’ occupation and implications for optimal feeding, cultural barriers, food preferences and availability of foods.

Extent of mothers’ interaction with LHWs and relationship with LHWs will be examined and preferred strategies for communication will be identified.

**Methods:** Mixed methods, largely qualitative but also supported by quantitative data will be used to assess readiness.

1. Household Survey:

Relevant indicators will be built into the baseline household survey (described above), on the following areas:

- Presence of LHW
- Willingness to be enrollment for pre-prepared food supplement

1. Key Informant Interviews:

Interviews will be conducted with district, union council level key informants to inform on willingness and acceptability of program; operational feasibility; proposed delivery and accountability systems; and measures to enhance uptake in the community. Interviews will be conducted with the following proposed stakeholders:

- Nutrition Focal Person, District Health Office, Thatta
- Local Focal Person, World Food Program, Thatta
- Local Focal Person, Merlin, Thatta
- Representative of Food Department, Thatta
- District Health Officer, Thatta
- District Coordinator, LHW Program, Thatta
- Union Council Nazim, (6 interviews - Intervention Union Councils)
- Lady Health Supervisors (6 interviews -Intervention Union Councils)
- Local CSOs, Thatta (3-4 interviews)
- Private sector traders
- Transporters
- Food producers
- Agricultural extension workers
- Teachers
- Local water committees

Key informants at district level will be purposefully selected following the above list, and further interviewees will be selected using snowballing approach. Stakeholders from Union Councils will be drawn from 6 randomly selected intervention Union Councils. A semi-structured questionnaire will be used to elicit open ended responses on identified areas. Transcripts will be uploaded in NVivo for analysis.

C. Focus Group Discussions (FGDs)

Focus Group Discussions will be conducted with participants from 6 villages in the proposed intervention area. 6 intervention Union Councils will be randomly selected and then one village will be randomly selected from each Union Council.

Focus Groups Discussions will be held with a number of grassroots level stakeholders to provide a triangulated perspective of locally perceived barriers and solutions. 12-15 participants will be recruited for each FGD. These are as follows:

- Village elders - 1 FGD
- Lady Health Workers – 1 FGD
- Pregnant & Lactating Mothers, Mother-in laws- 6 FGDs
- Spouses of Pregnant & Lactating Mothers – 6 FGDs

Data collectors (note takers and moderators) will be recruited from Thatta with command of local language. Male data collectors will conduct FGDs with male participants and female data collectors with female participants. FGDs will be conducted after taking informed consent, and at convenient time and place for participants. A topic guide with probes will be used for an informed discussion, the moderator will facilitate free flow of discussion while note takers take notes of important verbal and non-verbal communications and gestures. FGDs will be tape recorded after taking consent from the participants. Transcription will be carried out on real time basis and uploaded into N-Vivo for thematic coding and analysis.

**II.** **Period Assessments:**

Roll out and uptake of essential interventions will be assessed six monthly.

A. Logistics Management Assessment

Delivery systems will be assessed six monthly in terms of supplies stock outs, adequacy of food storage systems, and record maintenance. This will be done through Direct Observation Checklist, and a review of information systems and record, with assistance of a procurement specialist from AKU.

1. Food Consumption at Community Level. Rapid six monthly assessments will be done at the village level to assess the following:

- Presence of LHW
- Frequency of interaction with LHW
- Proportion enrolled into the program amongst eligible population
- Mother’s knowledge of delivery points for food supplementation
- Proportion who received food supplements amongst those enrolled
- Mother’s knowledge of appropriate usage of food supplementation
- Frequency of food supplementation usage by mother
- Willingness to continue food supplementation usage

1. Uptake of Essential Interventions by Households:

This will include:

1. Provision of BCC through lane meetings, women’s groups, household sessions
2. Antenatal care visits to skilled provider
3. Handwashing
4. Provision and use of folic acid
5. Provision and use of iron
6. Provision and use of Multiple Micronutrient supplementation
7. Provision and use of food supplements
8. Purchase and use of iodized salt
9. Knowledge and practices of delayed cord clamping
10. Neonatal vitamin K administration
11. Vitamin A supplementation
12. Essential immunizations
13. Kangaroo mother care for promotion of breastfeeding and care of preterm and SGA infants

Infant and child feeding practices will also be assessed through rapid appraisal on six monthly bases:

- Early initiation of breastfeeding (within one hour of birth)
- Exclusive breast feeding under 6 months
- Continued breastfeeding at 1 year
- Introduction of solid, semi-solid or soft foods
- Minimum dietary diversity
- Minimum meal frequency
- Minimum acceptable diet (apart from breast feeding)
- Consumption of iron-rich or iron-fortified foods

**III. End line Assessment of Operational Aspects & Consequences of Food Based Intervention**

Assessment of implementation gaps and of systemic consequences other than stunting will be done at end line, using multi-pronged assessment at community, union council and district level. These findings will be used to inform decision to scale up.

*Programmatic gaps between design and implementation*

This section will explore synergies and constraints to program implementation through Household Survey, Distribution System Assessment, Key Informant Interviews and FGDs. It will assess the outreach of the program to intended beneficiaries in terms of enrollment, distribution and actual usage of food supplements. It will further assess the efficiency of distribution systems, adequacy of monitoring of food distribution and vulnerability for political capture and leakages. It will also look into product acceptability at community and district level probing what might have been improved in terms of product related features (taste, consistency etc), storage at household level, preferred distribution outlets and cultural or other constraints to use of food supplements. A particular focus will be on connects between the food and preventive health measures at the programmatic level and at the village level, examining the horizontal accountability of LHWs, ease of communication and the depth of information provided on food delivery, food consumption and accompanying health messages. All aspects will be examined with the perspective of where can future improvements be made.

*Unintended consequences including positive and negative outcomes*

Unintended consequences of food supplementation programs have not been well explored in developing country settings. Western countries have highlighted obesity in food stamp programs but this is less applicable to impoverished settings such as Pakistan. Open ended exploration of both positive and negative outcomes will be done at district, UC and village level using Key Informant Interviews and FGDs. Areas explored will include potential for financial dependency on food supplements, crowing out of local foods by prepared supplements, demand overtaking supply, and other negative outcomes. Unintended benefits, not captured in stunting measurements, will also be explored such as better community linkage with LHW Program, enhancement of women’s agency and other positive outcomes.

**Methods:** Mixed methods will be used relying on quantitative assessments supported by exploratory qualitative data.

1. Household Survey:

Relevant indicators will be built into the baseline household survey questionnaire (discussed in separate section), on the following areas:

- Presence of LHW
- Frequency of interaction with LHW
- Proportion enrolled into the program amongst eligible population
- Mother’s knowledge of delivery points for food supplementation
- Proportion who received food supplements amongst those enrolled
- Mother’s knowledge of usage of food supplementation
- Frequency of food supplementation usage by mother
- Willingness to continue food supplementation usage
- Mother’s knowledge on IYCF
- IYCF practices
- Provision of BCC through lane meetings, women’s groups, household sessions
- Antenatal care visits to skilled provider
- Handwashing
- Provision and use of folic acid
- Provision and use of iron
- Provision and use of Multiple Micronutrient supplementation
- Provision and use of food supplements
- Purchase and use of iodized salt
- Knowledge and practices of delayed cord clamping
- Neonatal vitamin K administration
- Vitamin A supplementation
- Essential immunizations
- Kangaroo mother care for promotion of breastfeeding and care of preterm and SGA infants

B. Logistics Management Assessment

Delivery systems will be assessed six monthly in terms of supplies stock outs, adequacy of food storage systems, and record maintenance. This will be done through Direct Observation Checklist, and a review of information systems and record, with assistance of a procurement specialist from AKU.

1. Key Informant Interviews

Interviews will be conducted with district, union council level key informants to inform on willingness and acceptability of program; operational feasibility; proposed delivery and accountability systems; and measures to enhance uptake in the community. Interviews will be conducted with the following proposed stakeholders:

- Provincial Program Director, Nutrition, Government of Sindh
- Local Focal Person, World Food Program, Thatta
- Local Focal Person, Merlin, Thatta
- Representative of Food Department, Thatta
- District Health Officer, Thatta
- District Coordinator, LHW Program, Thatta
- Union Council Nazim, (12 interviews - Intervention Union Councils)
- Lady Health Supervisors (12 interviews -Intervention Union Councils)
- Local CSOs, Thatta (3-4 interviews)
- Any other

Key informants at district level will be purposefully selected following the above list, and further interviewees will be selected using snowballing approach. Stakeholders from Union Councils will be drawn from 12 randomly selected intervention Union Councils. A semi-structured questionnaire will be used to elicit open ended responses on identified areas. Transcripts will be uploaded in NVivo for analysis.

D. Focus Group Discussions (FGDs)

Focus Group Discussions will be conducted with participants from 12 villages in the intervention area. 12 intervention Union Councils will be randomly selected and then one village will be randomly selected from each Union Council. Focus Groups Discussions will be held with a number of grassroots level stakeholders to provide a triangulated perspective of locally perceived barriers and solutions. 12-15 participants will be recruited for each FGD. These are as follows:

- Village elders - 2 FGD
- Lady Health Workers – 2 FGD
- Pregnant & Lactating Mothers and mother in -laws- 12 FGDs
- Spouses of Pregnant & Lactating Mothers – 12 FGDs

Data collectors (note takers and moderators) will be recruited from Thatta with command of local language. Male data collectors will conduct FGDs with male participants and female data collectors with female participants. FGDs will be conducted after taking informed consent, and at convenient time and place for participants. A topic guide with probes will be used for an informed discussion, the moderator will facilitate free flow of discussion while note takers take notes of important verbal and non-verbal communications and gestures. FGDs will be tape recorded after taking consent from the participants. Transcription will be carried out on real time basis and uploaded into N-Vivo for thematic coding and analysis.

**Cost Effectiveness Analysis**

Cost effectiveness analysis will run through the full three years of the program. For our analysis we will have 2 arms whose cost effectiveness will be compared: Arm 1: food based and preventive health interventions; Arm 2: preventive health interventions only. The time frame for both will be three years of intervention

To be able to assess the cost effectiveness of the intervention and control arms current, we will compare interventions with a scenario of doing nothing to improve child health. Incremental cost effectiveness ratios will be computed for each arm by calculating the incremental improvement over Do Nothing scenario.

A sensitivity analysis will be carried out to report results with or without 3% discounting for DALYs and using cost inputs for foreign prepared versus locally prepared supplements.

*Cost Calculation:* Unit costs for provision of food supplements and for delivery of preventive health measures will be developed based on programmatic records. A record of program inputs and costs will be maintained during the length of the program and verified by an external accountant. 2013 as the base year will be kept as the base year with costs converted to 2013 purchasing power parity to remove the effect of inflation. All costs will be summarized in US dollars and future costs discounted at 3%.

*Effect Calculation*: Percent reduction in stunting will be taken as the effect outcome. Efficacy will be obtained through Before After household survey. Changes in linear growth will also be calculated.

**Team Composition: Operational Design, Unintended Consequences and Cost Component**

- Co-Principal Investigator and Research Supervisor will be responsible for technical design, oversight on field component, analysis and report write-up.
- Research Coordinator will be responsible for overseeing all field activities, data processing and assistance with data analysis.
- Social Scientist will supervise qualitative data collection for Key Informant Interviews and FGDs assisted by Research Assistants from the field. Procurement Specialist will assess the delivery systems, and a chartered accountant will maintain program costs and compute unit costs.
- An Administrative Assistant and Finance Officer will provide logistics, coordination and financial accounting support to the project.

**Confidentiality:**

Confidentiality of all the data collected from the population will be a high priority. All the names and personal information regarding any individual will not to be disclosed and all the names present in the forms will be de linked and forms will be coded accordingly. Only senior level staff will have the access to the data. Participant privacy and confidentiality in electronic and printed data, publications, and reports during and following completion of the survey will be ensured.

**Ethical Considerations:**

The project doesn’t depict any major ethical considerations. The project proposal will be submitted to the Ethics review committee of Aga Khan University and National Bio ethics committee (NBC) of Pakistan Medical and Research Council PMRC for review and approval.

**Data ownership and Publication Policy**

The data in both hard and soft form will be joint intellectual property of AKU and WFP. The final data sets with computing variables will be shared between Health department, WFP and AKU. AKU will develop the first draft report of the study activities. AKU will publish the data with the mutual consensus of WFP in peer reviewed journals.

**Dissemination of Findings and Future Plans**

The cleaned dataset along with overall analyses will be provided to WFP and Ministry of Health. WFP in collaboration with Department of Health Sindh and AKU and other collaborative partners will disseminate the findings of the results once the report is finalized.

**Citations:**

Government of Pakistan (2011): National Nutrition Survey (NNS), 2011. Planning Commission Planning and Development Division Government of Pakistan.

WFP (2012): Protracted Relief and Recovery Operations (PRRO). Pakistan 200250. Rome, Italy.

Horton, S. 1999: 0pportunities for investments in nutrition in low-income Asia. Asian Development Review 17 (1,2): 246-273.

1. Grossman, Jean Baldwin. 1994: [Evaluating Social Policies: Principles and U.S. Experience](http://www-wds.worldbank.org/servlet/WDSContentServer/WDSP/IB/1999/09/25/000178830_98101911372689/Rendered/PDF/multi_page.pdf). The World Bank Research Observer 9(2): 159-80. Baker J, 2000; Prennushi, et al, 2000 World Bank Report.
2. Gorstein J, Sullivan KM, Parvanta I, Begin F: Indicators and Methods for Cross-Sectional Surveys of Vitamin and Mineral Status of Populations. The Micronutrient Initiative (Ottawa) and the Centers for Disease Control and Prevention (Atlanta), May 2007.
3. Donner, A. and Klar, N. 2000: Design and Analysis of Cluster Randomization Trials in Health Research. Arnold, London.

1. Lipid- based nutrient supplement [↑](#footnote-ref-1)
2. Chick peas based type [↑](#footnote-ref-2)
3. Micronutrients powders [↑](#footnote-ref-3)
