# Supplementary material for: Effect of lipid-based nutrient supplement—Medium quantity on reduction of stunting in children 6-23 months of age in Sindh, Pakistan: A cluster randomized controlled trial
Source: PLoS One. 2020 Aug 13;15(8):e0237210. doi: 10.1371/journal.pone.0237210 (PMC7425934; doi:10.1371/journal.pone.0237210)
Supplement: S2 File — (DOCX) [file pone.0237210.s002.docx]

PONE-D-19-15573R3
Effect of lipid-based nutrient supplement - medium quantity on reduction of stunting in children 6-23 months of age in Sindh, Pakistan: a cluster randomized controlled trial

Dear Dr. Soofi:

Thank you for returning your manuscript and for your continued cooperation with our requests. There are still some outstanding issues that must be resolved before your manuscript can be formally accepted – please see below.

To access your manuscript and complete these changes, please follow this link: ********.

You will find the submission in “Current Task Assignments” and will need to click “Submit Task” in order to upload your corrected files. Your current files can be downloaded through the “Assignment Files” link; you can also download the manuscript file attached to this email.

Your task is due Jul 31 2020 11:59PM.

Response: Done

We appreciate your patience during this process. Please contact plosone@plos.org with any questions or concerns.

With kind regards,
PLOS ONE staff

JOURNAL REQUIREMENTS:


1. We note your Data Availability Statement currently reads as follows:

“De-identified data set will be uploaded after acceptance of the manuscript.”

1.) At this time, please provide the minimal anonymized data set necessary to replicate your study’s findings for inclusion as Supporting Information files, or upload your data set to a stable public repository. Please also provide the relevant URLs, DOIs, or accession numbers for any data uploaded to a repository. For a list of acceptable repositories, please see http://journals.plos.org/plosone/s/data-availability#loc-recommended-repositories.

2.) If you provide the minimal anonymized data set for inclusion as Supporting Information files, please also confirm whether we may update your Data Availability Statement as follows:

“All relevant data are within the manuscript and its Supporting Information files.”


Response: Attached minimal anonymized data set as supporting files. Yes please update data availability statement as above.

CONFIDENTIAL: This email and any attachments are confidential and for the sole use of the individual(s) to whom they are addressed. If you have received this message in error please delete the message and notify plosone@plos.org.

__________________________________________________
In compliance with data protection regulations, you may request that we remove your personal registration details at any time. (Use the following URL: https://www.editorialmanager.com/pone/login.asp?a=r). Please contact the publication office if you have any questions.
